# Multimodal Imaging Using Raman Spectroscopy and FTIR in a Single Analytical Instrument with a Microscope (Infrared Raman Microscopy AIRsight, Shimadzu): Opportunities and Applications

**DOI:** 10.3390/ijms25136884

**Published:** 2024-06-23

**Authors:** Kamil Jurowski, Maciej Noga, Damian Kobylarz, Łukasz Niżnik, Alicja Krośniak

**Affiliations:** 1 Laboratory of Innovative Toxicological Research and Analyzes, Institute of Medical Sciences, Medical College, Rzeszów University, Al. mjr. W. Kopisto 2a, 35-959 Rzeszów, Poland; 2 Department of Regulatory and Forensic Toxicology, Institute of Medical Expertises, ul. Aleksandrowska 67/93, 91-205 Łódź, Poland

**Keywords:** multimodal imaging, Raman spectroscopy, FTIR

## Abstract

Raman spectroscopy and Fourier transform infrared (FTIR) spectroscopy are powerful analytical techniques widely used separately in different fields of study. Integrating these two powerful spectroscopic techniques into one device represents a groundbreaking advance in multimodal imaging. This new combination which merges the molecular vibrational information from Raman spectroscopy with the ability of FTIR to study polar bonds, creates a unique and complete analytical tool. Through a detailed examination of the microscope’s operation and case studies, this article illustrates how this integrated analytical instrument can provide more thorough and accurate analysis than traditional methods, potentially revolutionising analytical sample characterisation. This article aims to present the features and possible uses of a unified instrument merging FTIR and Raman spectroscopy for multimodal imaging. It particularly focuses on the technological progress and collaborative benefits of these two spectroscopic techniques within the microscope system. By emphasising this approach’s unique benefits and improved analytical capabilities, the authors aim to illustrate its applicability in diverse scientific and industrial sectors.

## 1. Introduction

Fourier Transform Infrared (FTIR) spectroscopy is a critical analytical technique utilised across various scientific disciplines, including chemistry [1], biology [2], and environmental sciences [3,4]. FTIR spectroscopy is a crucial technique for characterising materials, including proteins [5] and carbon-based materials [6]. It offers a versatile, non-destructive, and relatively easy-to-use method for identifying chemical structures through their interactions with infrared radiation. FTIR spectroscopy’s operation principle is based on its ability to measure the absorption of infrared light by a sample, which causes molecular bonds within the sample to vibrate at specific frequencies. These frequencies are characteristic of the sample’s molecular structure, enabling the identification and characterisation of its chemical composition. FTIR spectroscopy typically covers the mid-infrared region, spanning from approximately 4000 to 400 cm⁻^1^ (2.5 to 25 micrometres (µm) in wavelength). This range covers the vibrational modes of most organic and inorganic compounds [7]. At the heart of FTIR is the mathematical technique known as Fourier transform. This technique converts a time-based signal into a frequency-based signal, enabling the identification of the individual frequencies (and, thus, chemical bonds) present in the sample. FTIR spectroscopy does not measure the spectrum by separating it into component wavelengths. Instead, it uses an interferometer to generate an interferogram, a complex signal resulting from the superimposition of all the different wavelengths in the infrared light after passing through the sample. Fourier transform is applied to this interferogram to reconstruct the absorption spectrum [8]. 

While a versatile and widely used technique, FTIR Spectroscopy has its disadvantages. Although FTIR spectroscopy is excellent for qualitative analysis, providing detailed information on chemical compositions, it has limitations in quantitative analysis. Quantifying the components of a mixture accurately requires extensive calibration and can be influenced by the matrix effect, where the presence of other substances affects the absorption characteristics of the analyte of interest [9]. Another limitation is the presence of water in the sample. Samples containing a significant proportion of water pose a challenge due to the extensive absorption of water, which requires special considerations to analyse aqueous solutions [10]. Moreover, the effectiveness of FTIR spectroscopy is highly dependent on the quality and preparation of the sample. Incorrect sample preparation can lead to inaccurate results, highlighting the technique’s sensitivity and the need for standardised procedures to ensure reliability and reproducibility [11]. 

Some of these limitations can be overcome by Raman spectroscopy. Fourier transform infrared (FTIR) spectroscopy provides insights into both organic and inorganic molecules, with a focus on identifying specific functional groups such as NH, OH, PO_4_^3−^, and CO_3_^2−^, among others [12]. On the other hand, Raman spectroscopy is utilized to analyse the skeletal vibrations within molecules. Raman and FTIR spectroscopy can provide complementary information. For instance, Raman is more sensitive to nonpolar bonds and symmetric vibrations, whereas FTIR excels in detecting polar bonds. This complementarity enhances the analysis of complex samples, such as biofluids, where polar and nonpolar components play crucial roles [13]. Raman spectroscopy is inherently nondestructive, allowing for the analysis of samples in their natural state without the need for elaborate preparation. This aspect is particularly beneficial in pharmaceuticals, where the sample’s integrity is crucial [14]. Raman spectroscopy’s reduced sensitivity to water makes it exceptionally suited for studying biological tissues, cells, and other water-containing samples without the interference that often plagues FTIR spectra. This advantage is critical in biomedical applications, where the natural hydration of samples is essential for accurate analysis [15]. The intrinsic advantages of Raman spectroscopy, such as its nondestructiveness, high resolution, and depth-profiling capabilities, have led to its widespread application across various fields. From the characterisation of pharmaceuticals [16] and polymers [17] to in-depth studies of biological samples [18] and advanced materials [19], Raman spectroscopy has proven to be a versatile and powerful analytical technique. Therefore, many laboratories use various FTIR and Raman spectrometers. However, this poses many problems, primarily due to the inability to analyse the same part of a sample with both. However, combining these two techniques in one device may provide new possibilities for synergistically measuring the same sample using both techniques [20]. The infrared/Raman microscope is an advanced analytical device uniquely combining infrared (IR) and Raman spectroscopy within a single compact unit. This integration allows for the measurement of both IR and Raman spectra from an exact sample location without the need to reposition, significantly enhancing the accuracy of analyses. This dual-spectroscopy setup for advanced imaging technology includes a proprietary wide-view camera that captures larger sample areas of up to 10 × 13 mm. This camera supports variable digital zooming, allowing for flexible adjustments to the field of view based on specific experimental needs. Furthermore, integrating this wide-view camera with a high-resolution microscope camera and objective lenses is critical for ensuring seamless transitions between different magnification levels and precise sample targeting. The system’s microscope camera can observe areas as small as 30 × 40 μm. For even greater detail, a 50× objective lens allows for observations of areas as small as 15 × 20 μm, and the 100× objective lens can achieve remarkable resolution, with the ability to view areas as minuscule as 7.5 × 10 μm. This meticulous attention to imaging detail is invaluable in various scientific fields, particularly in material science for the analysis of structural properties at the microscale, in pharmaceuticals for the detection of impurities or inconsistencies in drug formulations, and in environmental studies for the precise identification of microplastic pollutants. The ability of the system to provide such detailed images and data from the same location without physically adjusting the sample underscores its utility in advancing research and development in multiple disciplines [21].

This review article aims to comprehensively evaluate the capabilities and potential applications of Fourier transform infrared (FTIR) and Raman spectroscopy integrated within a single device. Specifically, this article explores the technical advances and synergies achieved by combining these two spectroscopic techniques under a microscope. By highlighting the distinct advantages of this hybrid approach and its enhanced analytical performance, the review seeks to demonstrate its utility across various scientific and industrial fields. Through a detailed examination of the microscope’s operation and case studies, this article illustrates how this integrated spectroscopic system can provide more thorough and accurate analysis than traditional methods, potentially revolutionising material characterisation.

## 2. Literature Review 

### 2.1. Opportunities 

Raman spectroscopy and infrared (IR) spectroscopy are classified under vibrational spectroscopy [22], a common technique used for the analysis of samples across the three fundamental states of matter [23]. The two techniques are mutually reinforcing and can offer more comprehensive insights into the sample than their individual use. When a combination of these techniques is used, one can obtain exact information about target compounds. This, in turn, facilitates the identification of analytes based on their diverse structural and biochemical characteristics [24]. Both methods are non-invasive and can examine delicate samples without destroying them or undergoing special preparation. Combining these two techniques allows researchers to access broader research possibilities. Infrared microscopy alone is insufficient for the identification of pollutants because they do not show high-intensity values in the spectrum [25,26]. However, the Raman spectrum can effectively determine the presence of a specific substance [27,28]. Analysis of organic substances can be performed using infrared microscopes, although obtaining information on a wide range of inorganic substances proves challenging. On the contrary, Raman microscopes offer the ability to acquire information about inorganic substances, such as titanium oxide and carbon, in addition to organic substances. Integrating these two techniques allows one to examine mixtures containing organic and inorganic compounds at a single measurement point, eliminating the need to relocate the sample [29]. 

Reports suggest that measuring racemic solutions (mixtures containing equal amounts of two enantiomers) using Raman spectroscopy is feasible [30,31,32,33], although it often poses significant challenges. However, combining Raman spectroscopy with infrared techniques offers a potential solution to this problem. Employing both spectroscopic methods makes it possible to identify racemic solutions and determine the correct enantiomer of the substance under examination. 

Combining two distinct spectroscopic techniques can enhance the certainty in identifying and characterising a test sample by corroborating the findings obtained from both approaches. Consequently, this leads to more resilient and reliable scientific conclusions. 

### 2.2. Applications 

#### 2.2.1. Microcontaminant Analysis 

Consumer concern about product contamination has increased the demand for analytical methods to address these concerns. Although media reports occasionally highlight contamination in certain medications and food items, completely eradicating this issue is challenging due to its diverse causes, including contamination of raw materials during procurement, contamination of products due to component degradation in production lines, and contamination by customers [34]. Furthermore, contaminants vary widely, including organic substances such as human hair, plastics, and rubber and inorganic materials such as oxides and metal fragments [35]. Therefore, enhancing the accuracy of qualitative analysis is necessary to pinpoint the source of contamination. An infrared microscope combined with a Raman instrument such as Shimadzu AIRsight, with a signal-to-noise ratio of 30,000:1, enables rapid acquisition of satisfactory spectra, even during microscopic contaminant analysis. The quality control of spectral analysis should be supported by specialised software that offers sample length measurement and spectrum advisory functions. An illustrative example involves measuring contamination on a button cell surface using an infrared microscope and analysing the contamination with a spectral database. 

**Figure 1:**
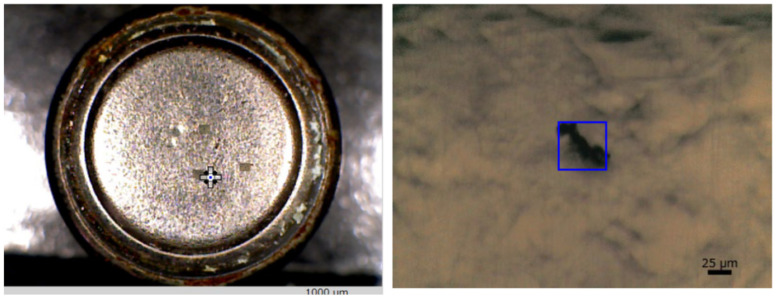
Appearance of contaminants on the surface of a button cell. **Left**: general observational image of the button cell captured by a wide-angle camera; **right**: observational image of contamination on the cell surface captured using a microscope camera [36].

Setting up a button cell on an infrared microscope stage and adjusting the microscope to ensure contamination measurement within the field of view (FOV) are time-consuming. Modern equipment features a wide-angle observation camera capable of surveying areas up to 10 × 13 mm and supporting variable digital magnification up to 5x (2.0 × 2.6 mm) to expedite this process. The wide-angle and microscope cameras provide position information, preventing FOV displacement due to camera changes. Observational images of microcontaminants on a button cell surface, captured by a wide-angle camera and a microscope camera, facilitate the location of adhering contaminants while assessing the overall cell image (Figure 1). 

**Figure 2:**
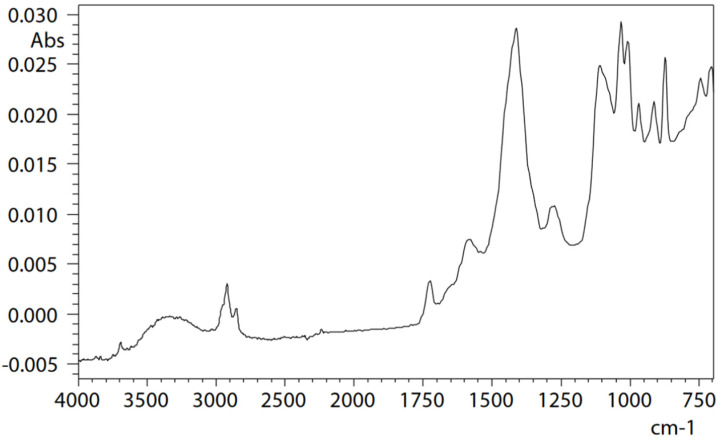
Infrared spectrum of a microcontaminant adhering to the button cell surface [36].

**Figure 3:**
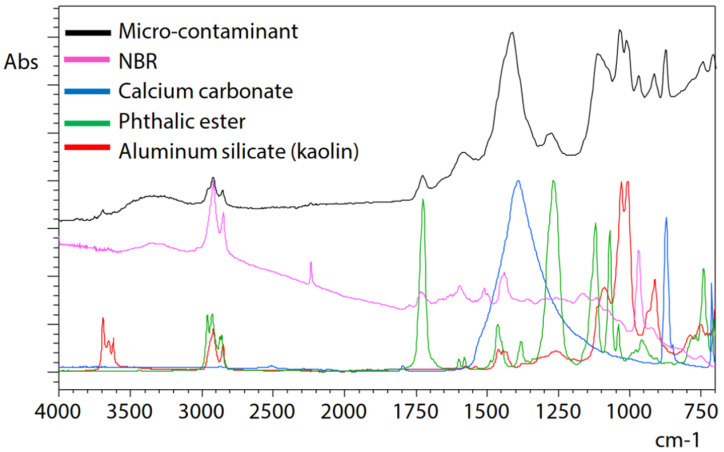
Result of the library search of the infrared spectrum [36].

ATR measurement of microcontamination adhering to the surface of a button cell [36] was conducted under the following measurement conditions: resolution, 8 cm^−1^; accumulation, 200; apodization function, SqrTriangle; detector, T2SL. Microcontamination was accurately captured and measured with high sensitivity, and a satisfactory, distortion-free infrared spectrum could be obtained. Therefore, it can be understood that microcontamination was captured precisely, noise was minimal, and high-sensitivity measurement was possible (Figure 2). Infrared spectral libraries were searched, and it was estimated that contamination consists mainly of acrylonitrile–butadiene rubber (NBR) as the main component, in addition to containing calcium, phthalate esters, and aluminium silicate (kaolin) as other additives (Figure 3). However, it should be noted that a thorough examination of each component is necessary when contamination consists of multiple components and experience in spectral analysis is required. 

Analysis of microcontaminants on a button cell surface using an infrared microscope emphasizes the utility of specialised spectral libraries in precise qualitative analysis during contamination and defect assessments. 

#### 2.2.2. Analysis of Microplastics 

**Figure 4:**
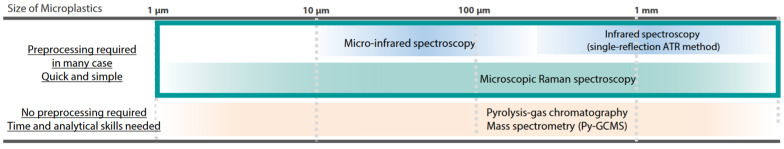
Methods for analysing microplastics by size [42].

The global spread of microplastic pollution in rivers and oceans raises concerns about its impact on aquatic life [37,38]. Exposure to UV radiation, rain, wind, and physical abrasion makes the plastic released into the environment brittle, breaking it into even smaller microplastic particles [39]. Typically, microplastics are assessed by examining their appearance, quantifying their abundance and size, and identifying their composition [40]. Among these criteria, determining the composition of the material is crucial for pinpointing the source of microplastics. However, as the size of the microplastics under evaluation decreases, the need for appropriate analytical tools becomes increasingly vital. A method to assess microplastics according to their size is described in Figure 4. Micro-Raman spectroscopy offers a means to analyse particles smaller than those detectable by infrared microspectroscopy and presents a more accessible alternative to pyrolysis–gas chromatography–mass spectrometry [41]. Integrating a Raman unit into an infrared microscope, as seen in the infrared/Raman microscope, facilitates both Raman and infrared analyses using a single instrument, streamlining processes that previously required separate equipment. 

Microplastics in water were sieved using polytetrafluoroethylene (PTFE) paper and accumulated on filter paper [42]. Since PTFE absorbs infrared only around 1200 cm^−1^, microplastics can be measured by transmission without removing the filter. After collection, the microplastics on the filter paper underwent infrared and Raman analyses using the infrared/Raman microscope. 

**Figure 5:**
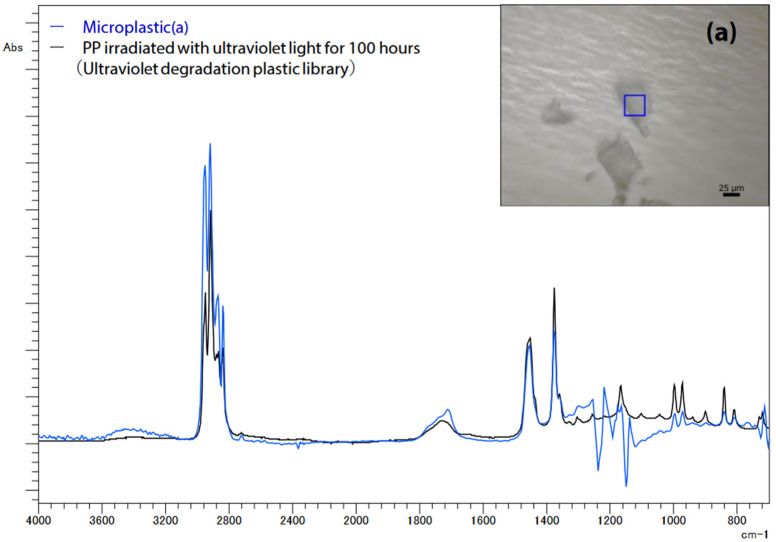
Infrared spectrum of microplastics (**a**) on filter paper [42].

Infrared light transmission microscopy was used to examine the microplastics (a) extracted from the filter paper. The measurement parameters included a resolution of 8 cm^−1^, an accumulation of 30 scans, Happ–Genzel apodization, a 25 μm aperture size, and a T2SL detector. Figure 5 illustrates the search results using the UV-damaged plastic library, revealing that microplastic (a) exhibited a spectrum similar to that of polypropylene (PP) irradiated with UV light for 100 h. The background noise around 1200 cm^−1^ is attributable to PTFE absorption, i.e., the filter paper’s material. 

**Figure 6:**
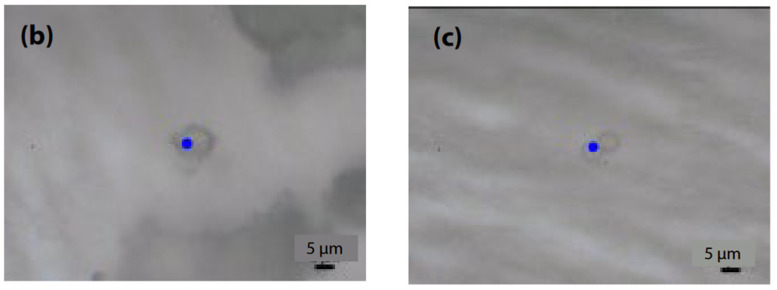
Images of microplastics (**b**) and (**c**) taken with an objective lens [42].

**Figure 7:**
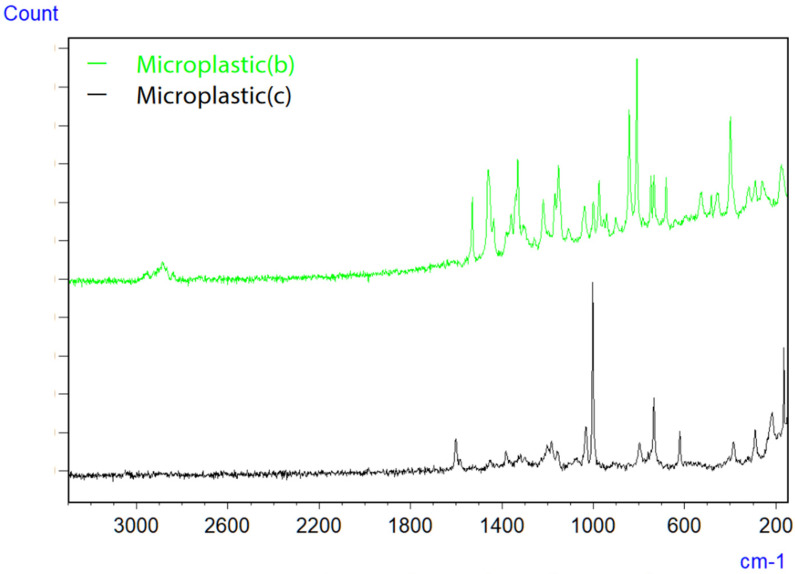
Raman spectra of microplastics (b) and (c) on filter paper [42].

Micro-Raman spectroscopy was employed to evaluate smaller microplastics, which are challenging to assess using infrared microspectroscopy due to the inability to focus light waves, as well as limited spatial resolution and detection capabilities. The images of microplastics (b) and (c) captured with the objective lens are depicted in Figure 6, along with the measurement parameters (an accumulation of 40 scans, an exposure time of 5.0 s, a 100x objective lens, an excitation wavelength of 785 nm, and a CCD detector), and the resulting Raman spectra are displayed in Figure 7. Although Raman spectroscopy typically operates at an excitation wavelength of 532 nm because of its strong Raman scattering, measurements were conducted at 785 nm to mitigate fluorescence interference. Consequently, microplastic (b) was identified as polyethylene (PE), while microplastic (c) was identified as polystyrene (PS). 

Microplastics of varying sizes were evaluated and categorised using an IR/Raman microscope. Although infrared microspectroscopy facilitates measurements of microplastics approximately 10 μm in size, combining it with micro-Raman spectroscopy enables the evaluation of microscopic samples below 10 μm, which were previously challenging to analyse solely via infrared microspectroscopy. 

#### 2.2.3. Evaluation of UV-Degraded Plastics 

In Raman spectroscopy, the scattering strength follows Rayleigh’s law, wherein it inversely correlates with the fourth power of the excitation laser’s wavelength [43]. Consequently, the intensity of the Raman signal depends on the wavelength of the laser used. Typically, shorter wavelengths are preferred to enhance signal strength, but this predisposition can also lead to fluorescence issues [44]. When a sample absorbs UV-visible radiation from a short-wavelength laser, it can emit fluorescence, which may obscure weaker Raman signals. In such scenarios, longer-wavelength lasers, which induce less fluorescence, can help alleviate its impact. An infrared Raman microscope equipped with standard 532 nm and 785 nm lasers empowers users to select the most suitable laser wavelength for their sample. The findings are based on a measurement using both lasers to assess UV-degraded plastics [45]. When plastics undergo UV radiation-induced damage, their spectral characteristics are altered, a phenomenon well documented in FTIR spectroscopy [46]. The examined plastics comprised nylon (polyamide, PA) and polyethylene (PE) measured both before exposure to UV radiation (unirradiated) and after exposure (irradiated) [45]. Raman spectroscopy was conducted under the following standardised conditions: accumulation, 10; exposure time, 5 s; objective lens, 50x; excitation wavelengths, 532 nm and 785 nm; detector, CCD. 

**Figure 8:**
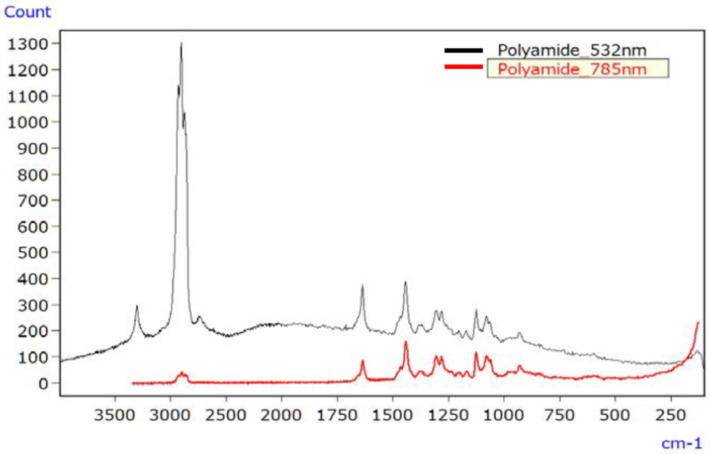
Raman spectra of unirradiated PA [45].

For PA, Figure 8 displays the Raman spectrum of the unirradiated sample using lasers with 532 nm and 785 nm wavelengths. Although discernible signal peaks were evident with the 532 nm laser (depicted by the black line), it resulted in a slightly elevated baseline due to fluorescence. Conversely, the spectrum obtained using the 785 nm laser (depicted by the red line) did not show such elevation. In particular, the wider range of wavenumbers measurable with the 532 nm laser facilitated the detection of various functional groups within the 4000 to 3000 cm^−1^ range. 

**Figure 9:**
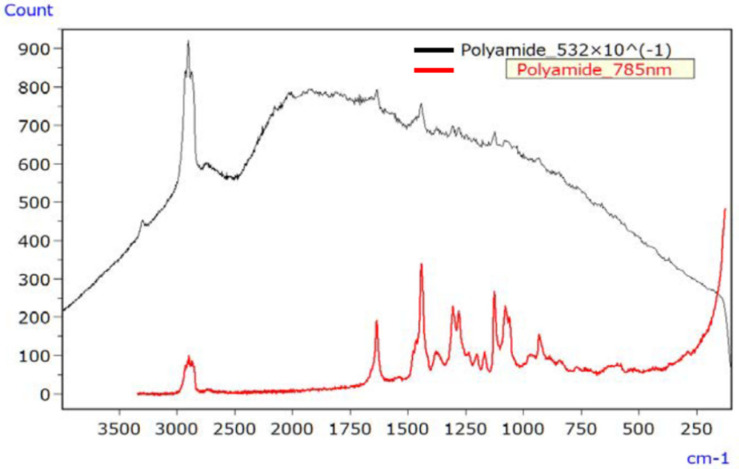
Raman spectra of UV-irradiated PA (532 nm data divided by 10) [45].

Figure 9 illustrates the Raman spectrum of the irradiated PA sample, employing lasers with wavelengths of 532 nm (black line) and 785 nm (red line). The 532 nm laser led to a significantly elevated baseline due to fluorescence, making signal peaks challenging to discern. In contrast, the spectrum obtained with the 785 nm laser exhibited minimal fluorescence interference and readily discernible signal peaks. 

**Figure 10:**
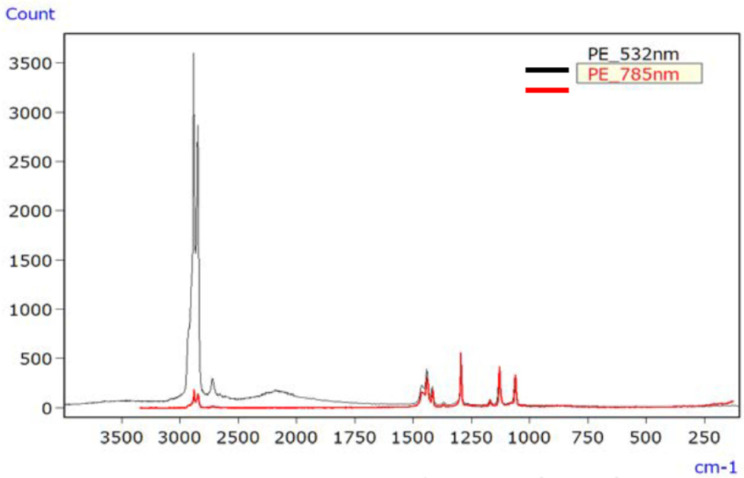
Raman spectra of unirradiated PE [45].

**Figure 11:**
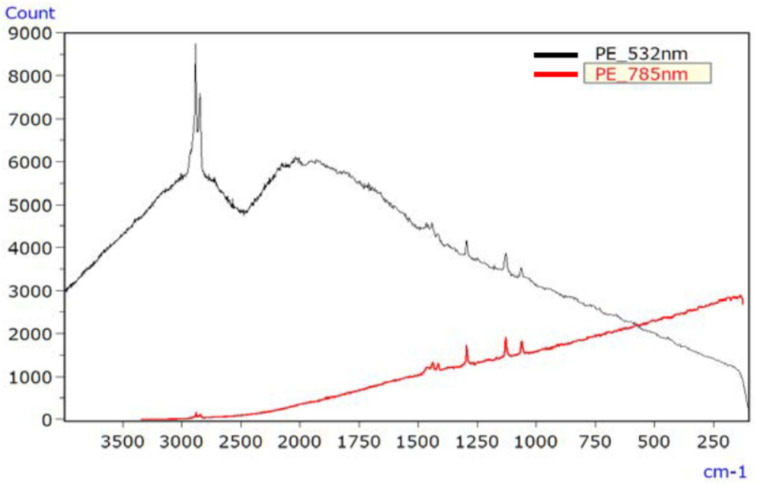
Raman spectra of UV-irradiated PE [45].

For PE, Figure 10 depicts the Raman spectrum of the unirradiated sample using lasers with wavelengths of 532 nm and 785 nm, with both lasers producing spectra devoid of raised baselines or discernible signal peaks. Figure 11 shows the Raman spectrum of the irradiated PE sample, where both lasers produced spectra with raised baselines due to fluorescence. In such cases, photo bleaching can be employed to mitigate fluorescence. 

Employing the 532 nm and 785 nm lasers of the infrared Raman microscope resulted in a reduction in fluorescence interference when using the 785 nm laser compared to the 532 nm laser. Additionally, fluorescence interference could be minimised by adjusting the photo-bleaching time. The microscope’s standard dual-laser configuration allows users to select the most appropriate laser for their sample and desired wavenumber range. 

#### 2.2.4. Analysis of Pigment Degradation 

Throughout history, pigments have served various purposes. Although natural inorganic pigments derived from minerals were commonly used in historical architectural paintings and murals, modern usage tends toward organic synthetic pigments due to their abundant availability and lower costs [47]. Identifying ancient and contemporary pigments requires the analysis of organic and inorganic substances, typically accomplished by infrared and Raman measurements, followed by data interpolation [48]. The advent of an infrared Raman microscope revolutionised this process by enabling the capture of both infrared and Raman spectra from a single spot using a single instrument, eliminating the need for multiple instruments and sample movement. Consequently, infrared and Raman spectra measurements of vermilion pigments and an analysis of their UV degradation were carried out [49]. The analysis of such materials requires minimal sample quantities, given the historical significance of pigments in the artwork. Ideally, a microscope capable of simultaneously measuring infrared and Raman spectra from a small spot would be utilised without sample movement. This would streamline the process, eliminating the need for multiple samplings and facilitating measurements with small sample quantities. Vermilion pigment was applied to wooden substrates with the following measurement conditions: infrared spectrophotometry (resolution: 8 cm^−1^; accumulation: 100; apodization function: SqrTriangle; detector: T2SL); Raman spectrophotometry (accumulation: 100; exposure time: 1.0 s; objective lens: 50x; excitation wavelength: 785 nm; detector: CCD). 

**Figure 12:**
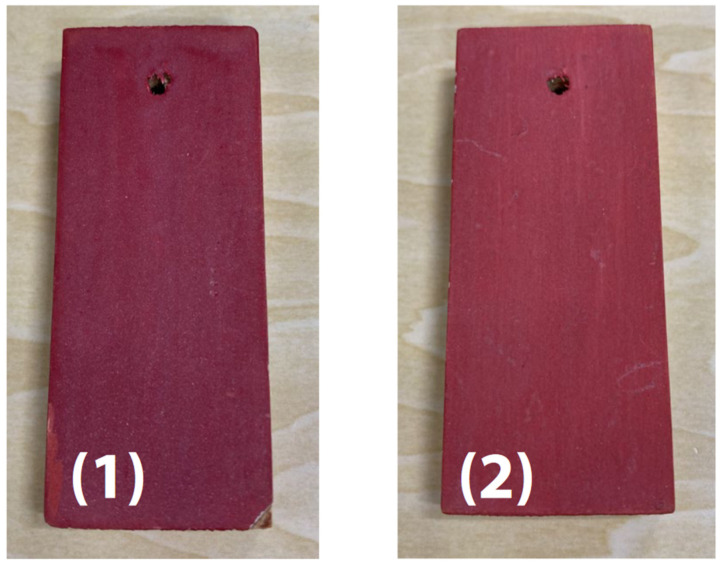
External appearance of vermilion pigment [49].

**Figure 13:**
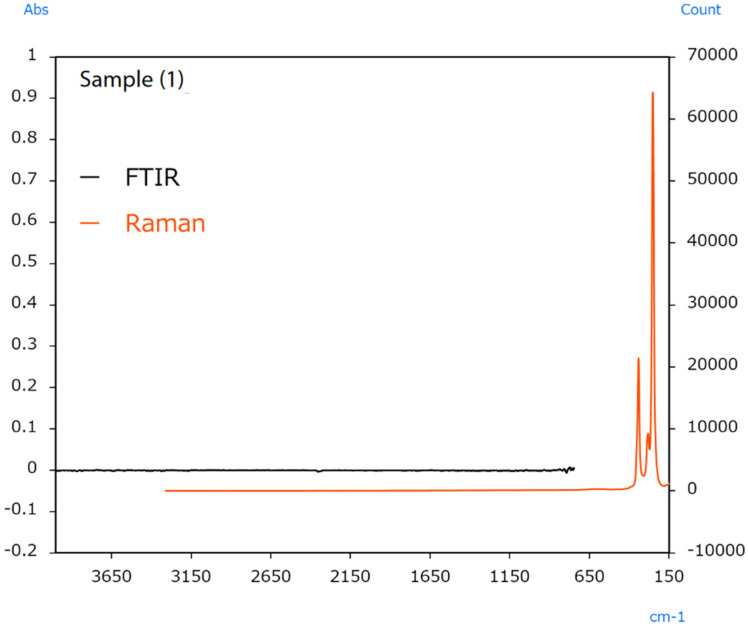
Infrared and Raman spectra of sample [49].

The sample’s surface (1) appeared irregular (Figure 12); therefore, infrared and Raman spectra were measured at several locations, but no differences in the spectra were observed between the locations. Typical infrared and Raman spectra are shown in Figure 13. 

Raman measurement results indicated peaks from HgS in the 370 to 185 cm^−1^ range. Vermilion is a pigment containing HgS that is known to have been used in ancient China [50]. The fact that no peaks were detected in infrared measurements performed by the ATR method of the microscope indicates a low probability that the sample contains organic substances. It was inferred that the only principal component is HgS. 

**Figure 14:**
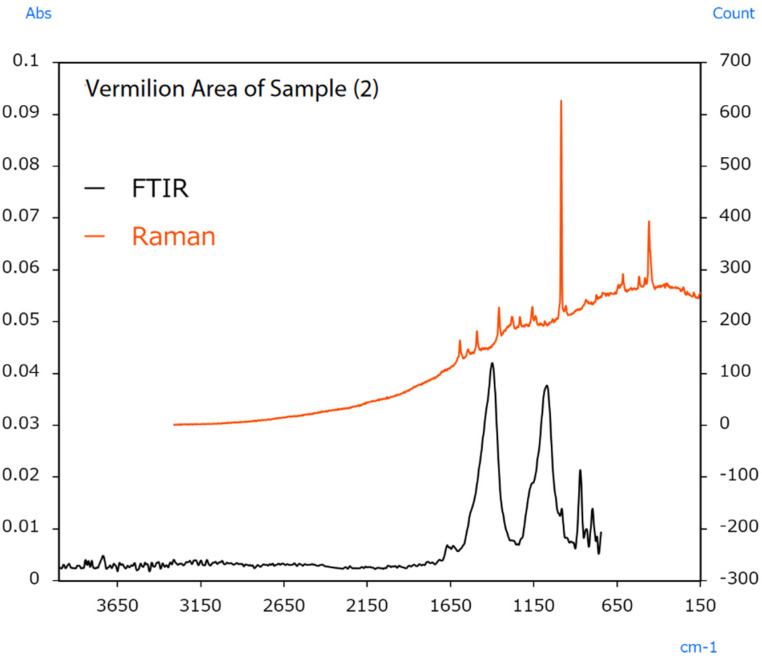
Infrared and Raman spectra of sample (2) (vermilion area) [49].

**Figure 15:**
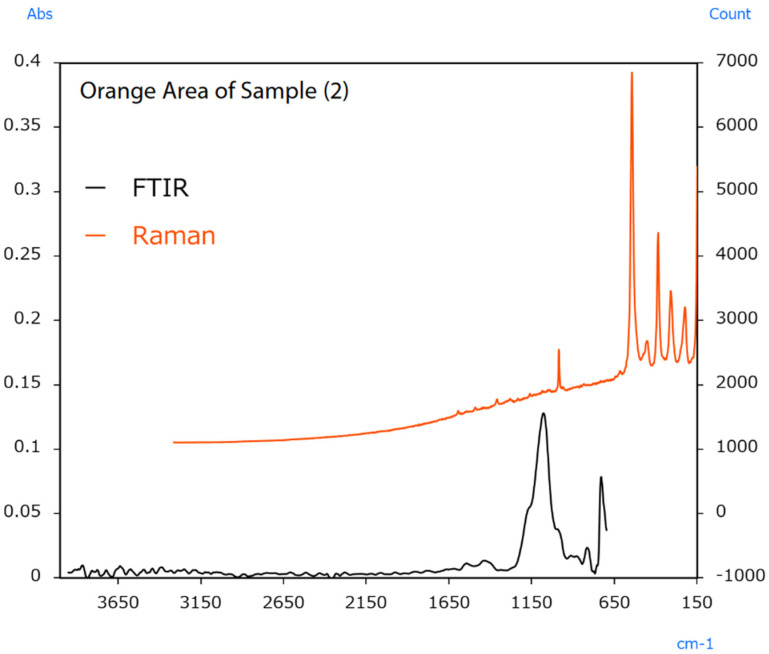
Infrared and Raman spectra of sample (2) (orange area) [49].

The sample’s surface (2) appears irregular, indicating colour differences between locations. In this case, infrared and Raman spectra were measured from the vermilion and orange areas. The resulting spectra are shown in Figure 14 and Figure 15. 

Raman measurement results from the vermilion area indicated a sharp peak from BaSO_4_ at 990 cm^−1^. Infrared measurement results indicated peaks from CaCO_3_ around 1400 cm^−1^. The results also indicated peaks from BaSO_4_ around 1050 cm^−1^. The BaSO_4_ and CaCO_3_ detected in these measurements were added to increase the amount of pigment or change its optical properties. Raman measurement results from the orange area indicated peaks from Pb_3_O_4_ in the 620 to 150 cm^−1^ range. Like HgS, Pb_3_O_4_ was also used as a vermilion pigment in ancient China [51]. The FTIR results also indicated the same BaSO_4_ peaks for the extender pigment as in the vermilion area. 

Raman spectrophotometry proved invaluable for identifying trace inorganic compounds, a challenging task for FTIR analysis. The study showcased the application of an infrared/Raman microscope in analysing vermilion pigment, offering the unique capability of capturing both infrared and Raman spectra from a single location using a single sample. This breakthrough allows for precise measurements, even from minute sample quantities, making microscopes particularly beneficial for analysing historically significant artefacts. 

While the combined infrared/Raman microscope demonstrates clear advantages, several factors warrant further consideration to maximize its utility. The precision in identifying both organic and inorganic components of pigments underscores its potential in conservation and restoration projects. However, the dependence on accurate spectral databases and the need for comprehensive calibration procedures cannot be overstated. Ensuring consistent and reproducible results across different studies is crucial for broader acceptance of this technology.

#### 2.2.5. Nondestructive Analysis of Diamond-Like Carbon (DLC) Film 

Diamond-like carbon (DLC) films consist of hard, amorphous layers made of carbon and hydrocarbons, representing a material that falls between graphite, which is known for its sp2 bonds, and diamond, which possesses sp3 bonds [52]. Due to their exceptional properties, DLC films have applications in various well-known products. For example, they are commonly used on the cutting edges of tools and bearing surfaces because of their outstanding resistance to wear and low-friction characteristics. Furthermore, DLC films are used on the inner surfaces of easily oxidisable beverage containers, exploiting their remarkable gas barrier properties [53]. Given the wide array of applications for DLC films, carbon bonding state and hydrogen concentration adjustments are made to tailor the film properties to specific requirements. However, measuring and regulating the bonding state and hydrogen concentration during the production or reception of DLC products is crucial because these factors can influence film properties. Raman spectroscopy is a quality control method for DLC films due to its ability to detect carbon materials’ bonding state and structure with high sensitivity [54]. Compared to X-ray photoelectron spectroscopy (XPS), another technique for DLC film analysis, Raman spectroscopy offers the advantages of easy preparation for measurements and nondestructive testing with minimal risk of sample damage [55]. Integrating an infrared/Raman microscope, which combines infrared and Raman microscopy, facilitates the analysis of various materials, including carbon materials, using a single instrument. This combination compensates for the strengths and weaknesses of each spectroscopic method and enables highly accurate qualitative analysis by measuring the same sample using both techniques. The Raman measurement capability of the infrared/Raman microscope has been used to examine DLC films deposited on silicon substrates [56]. Raman spectrum evaluation items for DLC films include the following:

- ZI(D)/I(G): Intensity ratio of the D band (around 1350 cm^−1^) and G band (around 1550 cm^−1^); disorder of the crystal structure (sp3/sp2 ratio) [57];
- FWHM (G): Half-width of the G band; crystallinity (sp2 bond), Young’s modulus, and density [54];
- log(N(G)/I(G)): Ratio of baseline and intensity at the position of the G band; hydrogen concentration [58].

**Figure 16:**
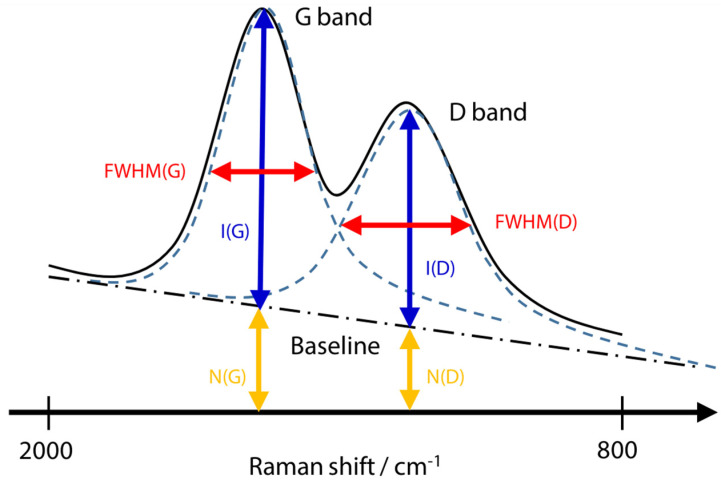
Schematic diagram of Raman spectrum evaluation items for DLC film [56].

Figure 16 shows the indices used here, where item I is the intensity from the corrected baseline to the top of the peak, N is the intensity from zero intensity to the corrected baseline, and FWHM is the half-width. 

I(D)/I(G) is a metric that indicates the crystal structure’s disorder level. In the Raman spectrum of DLC films, the G band (Graphite) typically emerges around 1550 cm^−1^, whereas the D band (Disordered) appears at approximately 1350 cm^−1^. The G band originates from vibrations of all sp2 bonds, including chain and cyclic varieties, whereas the D band arises from vibrations of sp3 bonds, indicative of disorder within the crystal lattice. Quantitative assessment of crystal structure disorder can be achieved by analysing the intensity ratio between the G and D bands obtained through wavelet separation of the DLC film’s Raman spectrum. The full width at half maximum of the G band (FWHM(G)) serves as an indicator of the crystallinity of sp2 bonds, with FWHM(G) typically increasing as the degree of amorphousness rises. Consequently, confirmation of FWHM(G) can evaluate the crystallinity of DLC films. Additionally, because it has been reported that FWHM(G) correlates positively with density and Young’s modulus, it can be inferred that this metric is effective for estimating the mechanical properties of films using a noncontact methodology. log(N(G)/I(G)) represents an indicator of hydrogen concentration. When Raman spectroscopy measures a DLC film containing hydrogen, the baseline will increase by an amount equivalent to the value of N (G) in Figure 16 due to the fluorescence effect. However, it is important to note that the fluorescence intensity is influenced by various arbitrary conditions, apart from hydrogen concentration, such as the laser intensity. The hydrogen concentration can be assessed by mitigating the influence of these arbitrary conditions through use of the ratio between the intensity of the Raman disorder (I(G)) and the intensity of the fluorescence component (N(G)). 

Two types of silicon substrates produced by DLC films were prepared as measurement samples [56]. In both cases, chemical vapour deposition (CVD) was carried out the film formation process, using CH_4_ or C_2_H_2_ as feedstock gases. To investigate potential changes in film quality, we measured the samples at two positions, namely near the centre and the outer edge. Measurement conditions are summarised as follows: accumulation, 100; exposure time, 1.0 s; objective: 50x; excitation wavelength, 532 nm; detector: CCD. 

Raman spectra were separated from the measured Raman spectrum using a curve-fitting function, and the quality of the DLC films was evaluated based on the obtained intensity values, half-widths, and other features. The intensity values were I(D)/I(G) = 0.32, FWHM(G) = 182.17, and log(N(G)/I(G)) = −0.29 for CH_4__center; I(D)/I(G) = 0.32, FWHM(G) = 181.40, and log(N(G)/I(G)) = −0.28 for CH_4__periphery; I(D)/I(G) = 0.34, FWHM(G) = 190.85, and log(N(G)/I(G)) = −0.44 for C_2_H_2__center; I(D)/I(G) = 0.34, FWHM(G) = 190.25, and log(N(G)/I(G)) = −0.44 for C_2_H_2__periphery. The appropriate evaluation metrics were consistent at both measurement positions for the CH_4_ and C_2_H_2_ feedstock gases used in this experiment. Consequently, the creation of uniform films without location-related discrepancies is deemed feasible. Notable variations, especially in FWHM(G) and log(N(G)/I(G)), were evident when samples were compared using different feedstock gases. From this observation, it can be inferred that the silicon substrate prepared using CH_4_ feedstock gas exhibited superior crystallinity to the substrate prepared using C_2_H_2_, and the hydrogen concentration in the CH_4_ film was higher. 

The ability to assess the quality of DLC films was showcased as an illustration of carbon material evaluation, underscoring the strength of Raman spectroscopy. Despite the variability in DLC film properties stemming from crystal structure disorder and hydrogen concentration, a quantitative evaluation of film quality was achievable through the use of micro-Raman measurement feature of the infrared/Raman microscope. Compared to alternative analytical methods, these measurements were nondestructive to the sample, and insights into the carbon bonding state and hydrogen concentration could be gleaned without the need for intricate sample preparation or measurement setups.

#### 2.2.6. Contaminant Analysis of Pharmaceuticals (Tablets) 

**Figure 17:**
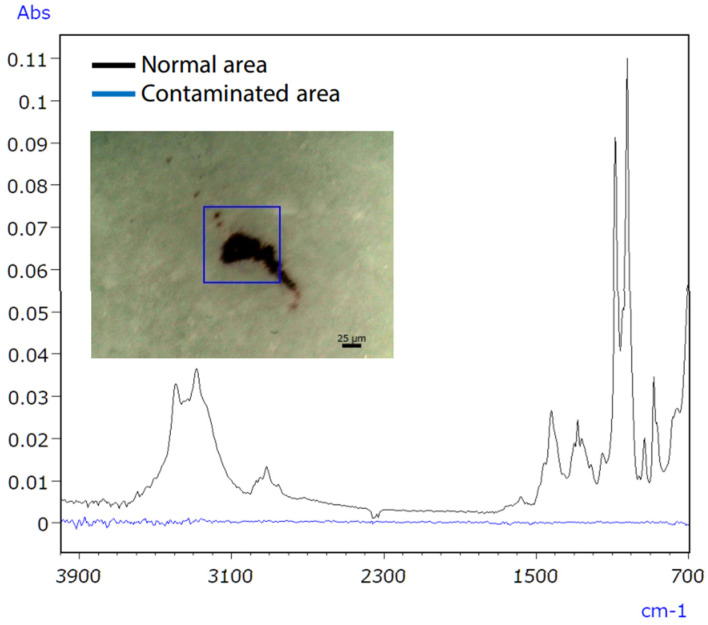
IR spectra of untainted and polluted tablet regions [61].

**Figure 18:**
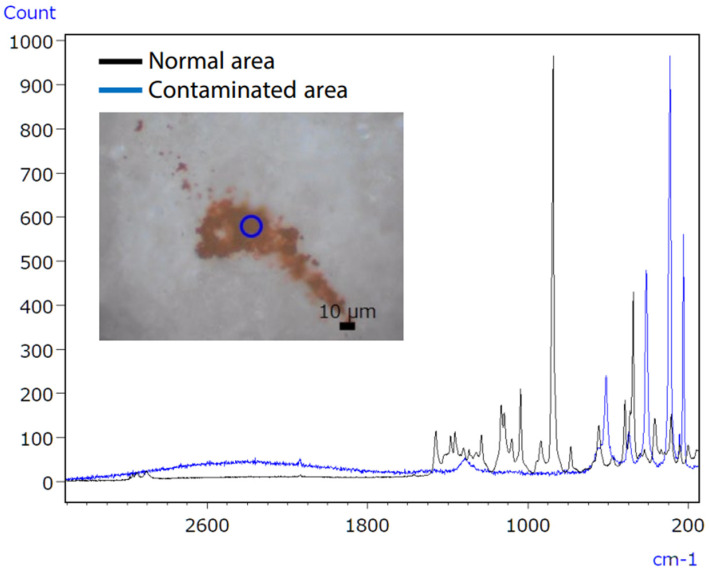
Raman spectra of uncontaminated and polluted tablet regions [61].

Consumer concerns about product contamination have recently increased, increasing the demand for analysis [59]. Eliminating contaminants in food and medicine is challenging due to the variety of sources, such as raw material contamination, production line errors, and consumer handling [60]. Hence, precise qualitative analysis is essential to accurately identify the sources of contamination. The infrared/Raman microscope, a novel instrument combining Raman and infrared analysis, addresses this need by enhancing accuracy in pinpointing contamination sources. Infrared spectroscopy might have difficulties with qualitative analysis of trace inorganic compounds, as shown in Figure 17. Raman spectroscopy successfully identified the inorganic contaminant, as presented in Figure 18. 

**Figure 19:**
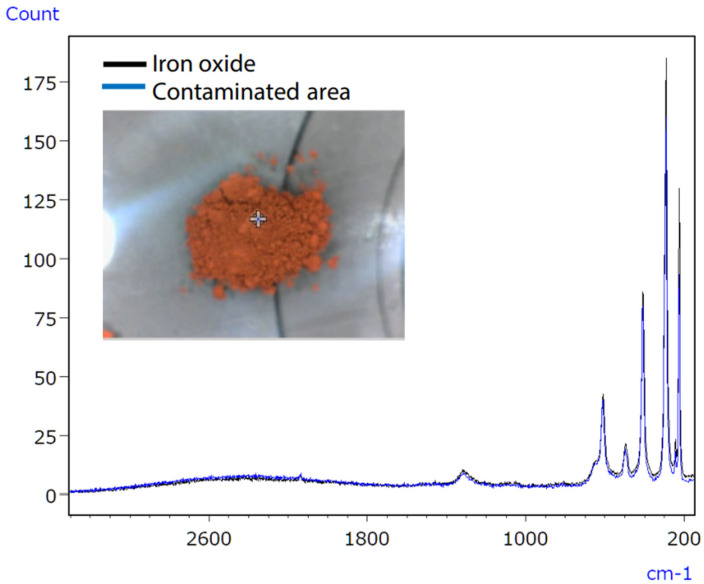
Raman spectra of the polluted tablet region and iron oxide [61].

Micro-infrared spectroscopy did not detect the characteristic peak of the contaminant, which was characterised as iron oxide (Figure 19), due to its position at a low wavenumber (510 cm^−1^). However, Raman spectroscopy proved effective in obtaining valuable data, as it possesses qualitative capabilities superior to those of inorganic compounds compared to infrared spectroscopy. The infrared/Raman microscope allows for seamless infrared and Raman spectrometry at an exact location with a single instrument. This feature makes it highly beneficial for qualitative analyses of unidentified samples. 

While the infrared/Raman microscope demonstrates significant advantages in contamination analysis, several factors should be considered to maximize its effectiveness. The ability of Raman spectroscopy to detect inorganic compounds at low concentrations is particularly valuable in ensuring product safety. However, the integration of Raman and infrared spectrometry into a single device also requires meticulous calibration and validation to ensure consistent and accurate results. Researchers and practitioners should focus on standardizing protocols and improving spectral databases to facilitate more reliable identification of contaminants.

#### 2.2.7. Rust Analysis 

Rust formation on metal surfaces consists primarily of metal oxides and hydroxides resulting from reactions between the metal, surface substances, and air [62]. Analysing these compounds can be complex and require specialised equipment, such as infrared spectroscopy with specific beam splitters and controlled environments. However, Raman spectroscopy offers a more accessible method that uses standard equipment to analyse the low-frequency regions of these compounds effectively. Additionally, energy-dispersive X-ray fluorescence spectrometry was used in this study, rendering it suitable for analysing single-element substances such as metals. 

**Figure 20:**
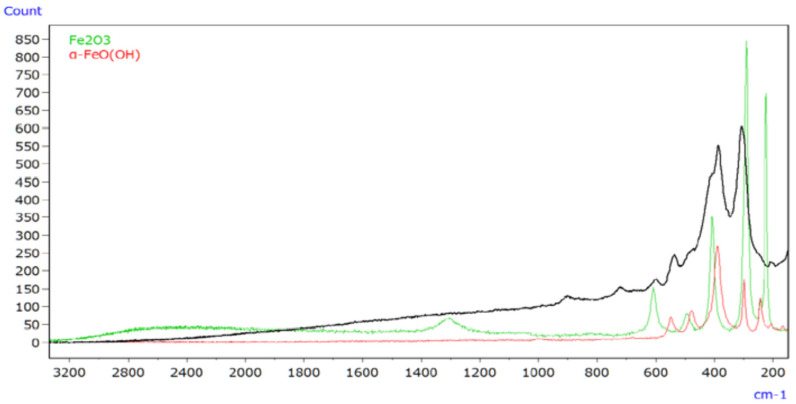
Raman spectra of rust (black line) and its constituents [63].

An infrared Raman microscope was used to analyse iron oxides and iron oxyhydroxides, the primary components of rust. Figure 20 represents Raman spectra of rust (depicted by the black line), α-FeO(OH) (illustrated by the red line), and Fe_2_O_3_ (represented by the green line). The study revealed that Raman spectroscopy can generate spectral profiles for inorganic compounds that are typically difficult to analyse using infrared spectroscopy [63]. Moreover, it highlighted that intense laser light can induce reduction reactions in inorganic compounds, altering the measured Raman spectrum. Additionally, real-world rust composition was determined by Raman spectroscopy, comparing its spectrum with those of candidate compounds or referencing a spectral database when candidate compounds are unknown. Furthermore, EDX analysis provided insights into metals undetected by infrared or Raman spectroscopy, detecting elements even at trace levels. 

While the study demonstrates the capabilities of Raman spectroscopy and EDX in rust analysis, several considerations merit further exploration. The ability of Raman spectroscopy to analyse inorganic compounds with high specificity highlights its potential in various fields, from cultural heritage conservation to industrial applications. However, the sensitivity of Raman spectroscopy to laser-induced changes in the sample composition necessitates careful calibration and control of experimental conditions. This is crucial to avoid misinterpretation of the spectral data, particularly in identifying the oxidation states of metals and the composition of complex oxides.

#### 2.2.8. Unstained Analysis and Evaluation of Bone Quality Characteristics of Rat Femur Cross Sections 

**Figure 21:**
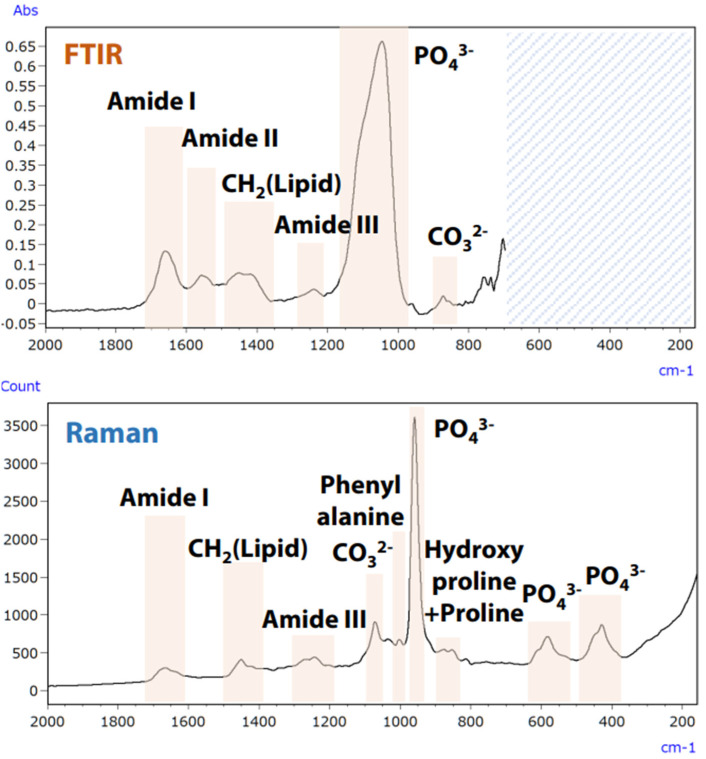
Analysis of rat femur using infrared and Raman techniques [66].

Preventing osteoporosis involves considering not only bone density but also bone quality, which includes properties such as the ratio of inorganic to organic components and the crystallinity of hydroxyapatite in bone tissue [64,65]. Micro-infrared and micro-Raman spectroscopy can evaluate these characteristics without staining bone samples [64]. These methods complement each other, with some information accessible only through one. An unstained analysis of a rat thighbone was performed using a specialist microscope that integrates both spectroscopy techniques, allowing for a comprehensive analysis and an optimal selection of methods for assessing bone quality characteristics [66]. Figure 21 shows the spectra and primary characteristics of the rat femur collected using both infrared (FTIR) and Raman spectroscopy. 

The integration of infrared and Raman spectroscopy enables simultaneous analysis of multiple components, such as collagen or hydroxyapatite, without moving the specimen. Furthermore, multivariate analysis allows for the acquisition of component spectra and chemical images of their distribution [66]. 

### 2.3. Analysis of Protein 

**Figure 22:**
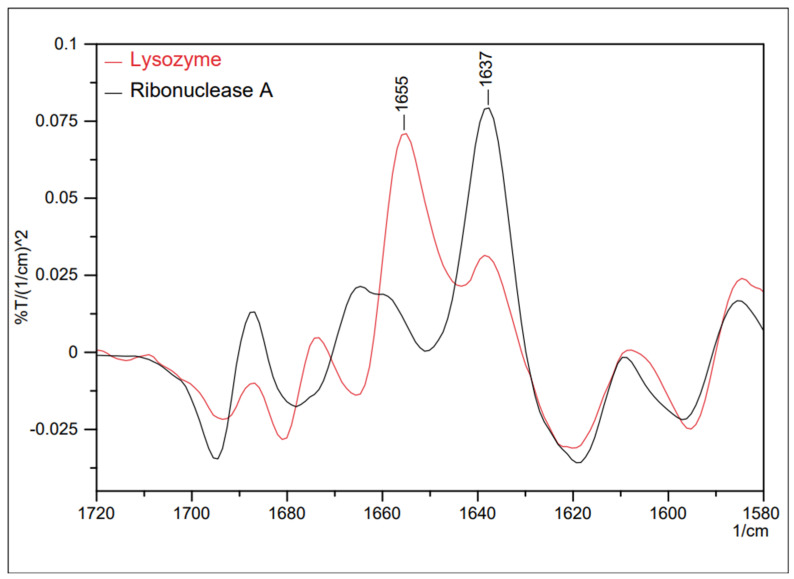
Secondary differential spectra of two proteins—lysozyme and ribonuclease A [70].

Proteins perform essential functions in living organisms, including signal transduction, genetic transcription, cellular apoptosis, immunity, structural reinforcement, and catalysis of chemical reactions. These processes are essential for ensuring the survival of organisms [67]. The importance of proteins is mainly associated with their conformations, which allow them to bind to other molecules appropriately [68]. FTIR spectroscopy examines elements affecting the secondary configuration, such as the α-helix and β-strand [69,70,71]. This is because the amide-I infrared bands of globular proteins exhibit prominent peaks in the wavenumber region below 1650 cm^−1^, serving as a dependable indicator of antiparallel β sheets [72,73]. These structures form through hydrogen bonding between the C=O and N-H groups of peptide bonds, either within individual polypeptide chains or between them. In water-based solutions, α helices absorb light around 1650 cm^−1^, β sheets absorb around 1630 cm^−1^, and loops absorb around 1645 cm^−1^ (with a broad absorption profile), as represented in Figure 22. It is evident from this that the lysozyme exhibits a significant presence of α helices, while ribonuclease A predominantly features β sheets. 

**Figure 23:**
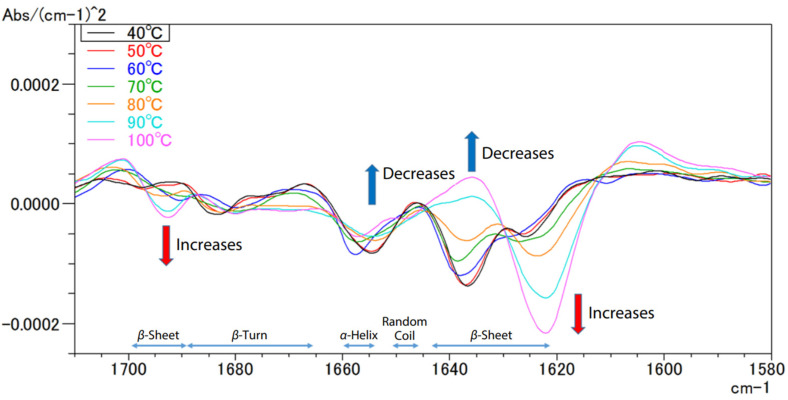
FTIR spectra of the second structures of the egg proteins at various temperatures [74].

FTIR can also be used to analyse changes during thermal denaturation [74]. This happens when a protein loses its original structure due to the weakening or disruption of weak chemical bonds and interactions, rendering it biologically inert [75]. Figure 23 visualises the second derivative spectra of egg white derived from the provided spectral data. 

The potential of FTIR to analyse proteins is widely recognised. It also extends to other molecules, such as total fat and carbohydrate levels, which may allow for analysis even in milk [76]. 

While the potential of FTIR spectroscopy is widely recognized, there are areas for improvement and innovation. One challenge is the interpretation of complex spectra, which can benefit from advanced computational tools and machine learning techniques. Additionally, FTIR’s sensitivity to environmental factors like hydration and pH can affect results, suggesting the need for methods to minimize these influences for reliable outcomes. Real-time monitoring of dynamic biological processes with FTIR is an underexplored area that could provide deeper insights into protein mechanisms, particularly in disease-related misfolding events. Combining FTIR with complementary techniques like Raman spectroscopy could enhance detection capabilities and offer a multi-dimensional view of biological samples. In summary, while FTIR spectroscopy is a powerful tool for protein analysis, addressing these challenges and exploring innovative approaches can further expand its applications and effectiveness in molecular biology.

## 3. Discussion

This review has explored the integration of Raman spectroscopy and Fourier transform infrared (FTIR) spectroscopy into a single, compact analytical device, highlighting the considerable advancements and synergies this combination provides for multimodal imaging. The dual spectroscopy system that integrates Raman and FTIR within a single unit provides a comprehensive molecular analysis by leveraging the strengths of both techniques. The sensitivity of Raman spectroscopy to nonpolar bonds and the capacity of FTIR to assess polar bonds allow for a more thorough and nuanced exploration of chemical structures. This integration enhances the accuracy of characterising complex mixtures, which is crucial in fields such as pharmacology, material science, and environmental science, where precise molecular distinctions are crucial. Table 1 shows the advantages and applications of IR and Raman spectroscopy.

The ability to conduct detailed analyses without extensive sample preparation is particularly beneficial for delicate samples susceptible to damage. The non-destructive nature of this integrated approach ensures that the integrity of the sample is maintained, which is essential for accurate and reliable data collection. This feature is vital in forensic analysis and heritage conservation, where sample preservation is as critical as the analysis itself. The integrated device is adept at handling a variety of samples, including but not limited to microplastics, pharmaceuticals, and complex biological matrices. The presented case studies demonstrate the system’s ability to discern fine molecular details in microcontaminants and microplastics, assess the quality of pharmaceuticals, and provide insight into the structural integrity of materials such as diamond-shaped carbon films. The practical applications of this technology are vast. In the pharmaceutical industry, for example, the ability to simultaneously use Raman and FTIR spectroscopy on the same microscopic regions of a tablet can drastically enhance the speed and accuracy of contaminant identification. Similarly, in environmental science, this technology allows for the precise detection and analysis of microplastics, a growing concern in aquatic ecosystems. The continued development and refinement of integrated Raman and FTIR systems is expected to open new avenues for analytical capabilities. Innovations such as increased automation, enhanced spatial resolution, and greater sensitivity will likely drive the adoption of this technology across more sectors. Additionally, expanding database libraries to include more substances and improving software algorithms for better peak identification will further enhance the utility and accuracy of these instruments. In conclusion, integrating Raman and FTIR spectroscopy into a single analytical instrument with a microscope setup represents a significant technological leap. This advancement improves the efficiency and effectiveness of analytical procedures and broadens the scope of potential applications, providing a powerful tool for researchers and professionals in a wide array of disciplines.

## 4. Materials and Methods

### 4.1. Search for Publications

The primary databases for finding published sources on this subject are Scopus, Google Scholar, and Web of Science. These were utilized to thoroughly analyse the main aspects related to topic of this review. It is important to note that besides these academic databases, additional sources, such as industry documents (especially manufacturer declarations), were also consulted during the data-gathering process.

### 4.2. Keywords and Selection of Scientific Data

Different combinations of the following main terms were used: multimodal imaging, single instrument, Raman spectroscopy, FTIR, opportunities, and applications. The study selection process involved two stages, namely (1) screening based on title and abstract and (2) thorough examination of full texts. Each author independently screened titles and abstracts at separate intervals. After defining the research problem, studies meeting the eligibility criteria proceeded to the next stage, while those clearly irrelevant or not meeting the exclusion criteria were excluded. All available sources, totalling 115 articles and related content, were analysed. Afterward, the authors carefully reviewed all the relevant manuscripts in full.

### 4.3. Presentation of the Results

To improve readability, we present the information in three sections. (1) The introductory segment discusses the basic principles of FTIR and Raman spectroscopy, explaining their respective methods and theoretical foundations. (2) The opportunities offered by combining FTIR and Raman spectroscopy are discussed in Section 2. This section explores how these methods complement each other, enhancing analytical capabilities and allowing for more detailed molecular characterisation. Specific examples illustrate the benefits of their synergistic use. Additionally, we provide more background and context to better introduce this part. (3) The range of potential applications for both analytical methods, showcasing various fields where they can be effectively applied is explored in Section 3.

## Figures and Tables

**Table 1:** Advantages and applications of IR and Raman spectroscopy for material analysis.

Feature	IR Spectroscopy	Raman Spectroscopy
Principle of Operation	Absorption of infrared radiation by bonds	Scattering of laser light (Raman scattering)
Spectral Range	4000–400 cm^−1^	4000–50 cm^−1^ (depending on the system)
Sample Requirements	Must be transparent in IR or thin	Can be transparent, opaque, liquid, or solid
Sample Preparation	May require special preparation, such as KBr pellets, depending on the used accessory, such as Seagull	Minimal preparation, often none required
Sensitivity to Water	Highly sensitive to water and moisture	Less sensitive to water and can analyse aqueous samples
Spectral Resolution	Limited by IR wavelength	High; can analyse small energy differences
Interferences	Problems with interferences from water and CO_2_	Fewer interference issues
Analysis Time	Short	Short
Qualitative Analysis Applications	Very effective in identifying functional groups	Excellent in identifying chemical compounds
Quantitative Analysis Applications	Possible, especially with FTIR techniques	Possible but less common
Applications	Polymers, pharmaceuticals, air pollutants, and mineral identification	Polymers, semiconductors, biochemistry, and pigment identification in art
Cost of Equipment	Relatively lower	Relatively higher
Advantages	Direct identification of functional groups and lower equipment cost	Minimal sample preparation and can analyse aqueous samples with high spectral resolution

## Data Availability

The data, analytic methods, and study materials that support the findings of this study are available from Kamil Jurowski (toksykologia@ur.edu.pl) upon reasonable request.

## Abbreviations

ATR	Attenuated Total Reflectance
CCD	Charge-Coupled Device
CVD	Chemical Vapor Deposition
DLC	Diamond-Like Carbon
EDX	Energy-Dispersive X-ray Spectroscopy
FTIR	Fourier Transform Infrared Spectroscopy
FWHM	Full Width at Half Maximum
IR	Infrared spectroscopy
NIR	Near-Infrared
PTFE	Polytetrafluoroethylene
SERS	Surface-Enhanced Raman Scattering
T2SL	Type-II Super Lattice
UV	Ultraviolet
XPS	X-ray Photoelectron Spectroscopy
